# Multiple Psychopharmacological Effects of the Traditional Japanese Kampo Medicine Yokukansan, and the Brain Regions it Affects

**DOI:** 10.3389/fphar.2017.00149

**Published:** 2017-03-21

**Authors:** Kazushige Mizoguchi, Yasushi Ikarashi

**Affiliations:** Tsumura Research Laboratories, Kampo Scientific Strategies Division, Tsumura & Co., IbarakiJapan

**Keywords:** yokukansan, Kampo medicine, multiple psychopharmacological effects, responsible brain regions, behavioral and psychological symptoms of dementia (BPSD)

## Abstract

Yokukansan (YKS), a traditional Japanese Kampo medicine, has indications for use in night crying and irritability in children, as well as neurosis and insomnia. It is currently also used for the remedy of the behavioral and psychological symptoms of dementia (BPSD), such as aggressiveness, agitation, and hallucinations. In parallel with clinical evidence, a significant amount of fundamental researches have been undertaken to clarify the neuropsychopharmacological efficacies of YKS, with approximately 70 articles, including our own, being published to date. Recently, we reviewed the neuropharmacological mechanisms of YKS, including its effects on glutamatergic, serotonergic, and dopaminergic neurotransmission, and pharmacokinetics of the ingredients responsible for the effects. This review is aimed to integrate the information regarding the psychopharmacological effects of YKS with the brain regions known to be affected, to facilitate our understanding of the clinical efficacy of YKS. In this review, we first show that YKS has several effects that act to improve symptoms that are similar to BPSDs, like aggressiveness, hallucinations, anxiety, and sleep disturbance, as well as symptoms like tardive dyskinesia and cognitive deficits. We next provide the evidence showing that YKS can interact with various brain regions, including the cerebral cortex, hippocampus, striatum, and spinal cord, dysfunctions of which are related to psychiatric symptoms, cognitive deficits, abnormal behaviors, and dysesthesia. In addition, the major active ingredients of YKS, geissoschizine methyl ether and 18β-glycyrrhetinic acid, are shown to predominantly bind to the frontal cortex and hippocampus, respectively. Our findings suggest that YKS has multiple psychopharmacological effects, and that these are probably mediated by interactions among several brain regions. In this review, we summarize the available information about the valuable effects of a multicomponent medicine YKS on complex neural networks.

## Introduction

The traditional Japanese Kampo medicine Yokukansan (YKS) has been approved by the Japanese Ministry of Health, Labor, and Welfare, and has indication for neurosis, insomnia, and children’s night crying and irritability. In recent year, this remedy has been used for treating the behavioral and psychological symptoms of dementia (BPSD), such as aggressiveness and hallucinations, which are frequently observed in patients with Alzheimer’s disease, dementia with Lewy body, vascular dementia, and so on, without a serious side effect (Iwasaki et al., 2005; Mizukami et al., 2009; Monji et al., 2009; Okahara et al., 2010; Nagata et al., 2012; Matsuda et al., 2013). YKS is also applied for symptomatic therapy of borderline personality disorder (Miyaoka et al., 2008a), tardive dyskinesia (Miyaoka et al., 2008b), treatment-resistant schizophrenia (Miyaoka et al., 2009), Asperger’s disorder (Miyaoka et al., 2012), postoperative delirium (Saito et al., 2010), preoperative anxiety (Arai et al., 2014), and neuropathic pain (Nakamura et al., 2009).

More recently, to facilitate greater understanding of the neuropharmacological mechanisms underlying the clinical efficacy of YKS, we reviewed the findings of basic studies. Our research indicated that YKS has multiple neurotransmitter mechanisms related to glutamatergic, serotonergic, cholinergic, dopaminergic, adrenergic, and GABAergic neurotransmission, and has neuroprotective effects and promoting effects on neuroplasticity including neurogenesis (Ikarashi and Mizoguchi, 2016). This suggested that YKS has multiple neuropharmacological actions, which might be involved in the various clinical benefits of YKS. Basic research has also shown the psychopharmacological effects of YKS against BPSD-like symptoms, other neurological symptoms, and cognitive deficits. For example, YKS has been demonstrated to ameliorate aggressiveness (Ikarashi et al., 2009; Iizuka et al., 2010), hallucinations (Egashira et al., 2008; Ueki et al., 2015a,b), anxiety (Mizoguchi et al., 2010b; Shoji and Mizoguchi, 2013), dyskinesia (Sekiguchi et al., 2012), neuropathic pain (Suzuki et al., 2012), and memory impairment (Ikarashi et al., 2009; Tabuchi et al., 2009; Fujiwara et al., 2011; Nogami et al., 2013; Uchida et al., 2013; Liu et al., 2014).

In this review, we aim to consolidate our understanding of the relationship between the psychopharmacological effects of YKS and the brain regions responsible for them. First, the psychopharmacological effects of YKS are described, as obtained primarily from animal studies, to show that YKS has multiple psychopharmacological effects. Because these effects are generally associated with brain regions responsible for specific functions, we next looked at the relationship between the psychopharmacological effects of YKS and the effects in different brain regions. Finally, the present findings were integrated into our understanding of the neuropharmacological mechanisms of YKS. Ultimately, we aim for this review to be utilized as a foundation for understanding the multiple psychopharmacological actions of YKS on complex brain networks (as circuits or pathways interlinking several brain regions) related to dementia and BPSD.

## Methodology

Manuscripts published from 2005 to 2016 describing the clinical efficacy and psychopharmacological effects of YKS were selected from PubMed database, the search term was “yokukansan”. Articles related with physiology, pathophysiology, and animal models of diseases were picked out manually. We excluded the articles that were written in a language other than English.

## YKS Sourcing and Standardization

Yokukansan is composed of seven dried medicinal herbs; Atractylodes lancea rhizome (4.0 g, rhizome of *Atractylodes lancea* De Candolle, *Compositae*), Poria sclerotium (4.0 g, sclerotium of *Poria cocos* Wolf, *Polyporaceae*), Cnidium rhizome (3.0 g, rhizome of *Cnidium officinale* Makino, *Umbelliferae*), Uncaria hook (3.0 g, thorn of *Uncaria rhynchophylla* Miquel, *Rubiaceae*), Japanese Angelica root (3.0 g, root of *Angelica acutiloba* Kitagawa, *Umbelliferae*), Bupleurum root (2.0 g, root of *Bupleurum falcatum* Linné, *Umbelliferae*), and Glycyrrhiza (1.5 g, root and stolon of *Glycyrrhiza uralensis* Fisher, *Leguminosae*). Identification for each plant material and preparation of YKS extract have been described previously (Ikarashi and Mizoguchi, 2016). Three-dimensional high-performance liquid chromatography has identified at least 25 ingredients in the methanol fraction of YKS extract (Ikarashi and Mizoguchi, 2016).

## Psychopharmacological Effects of YKS

### Effects of YKS on BPSD-Like Symptoms

#### Aggressiveness and Sociality

Oral administration of YKS ameliorated increased aggressiveness and decreased social behaviors in transgenic mice expressing human amyloid precursor protein (hAPP) (Fujiwara et al., 2011), rats and mice subjected to thiamine deficiency (Ikarashi et al., 2009; Iizuka et al., 2010; Yamaguchi et al., 2011), and serotonin (5-HT)-deficient rats (Kanno et al., 2009), as measured by social interaction tests. Further, YKS was shown to reduce the enhanced aggression of zinc-deficient mice in resident-intruder test (Takeda et al., 2012), as well as that of socially isolated mice, as measured by the reaction to a wooden stick (Uchida et al., 2009) or social interaction test (Nishi et al., 2012). In other research, YKS ameliorated increased aggressive behavior in mice intracerebroventricularly injected with amyloid β (Aβ) protein without physical inhibition, whereas antipsychotics reduced aggressive behavior but with concomitant suppression of motor activity (Sekiguchi et al., 2009). They also demonstrated that co-administration of YKS and donepezil ameliorated aggressive behavior in the same animal model, with donepezil not lessening the anti-aggressive effect of YKS, and YKS not interfering with the inhibitory effect on acetylcholinesterase activity and the improving effect on cognitive disturbance of donepezil (Sekiguchi et al., 2011). These agents may, therefore, have independent and complementary therapeutic effects on multiple dementia symptoms.

#### Hallucinations

Several hallucinogenic compounds such as lysergic acid diethylamide, *N*,*N*-dimethyltryptamine, and bufotenine, all with an indoleamine structure resembling 5-HT, have been demonstrated to induce the head-twitch response in animals (Nichols, 2004). In addition, agonistic action on central 5-HT_2A_ receptors evokes head-twitch (Lucki et al., 1984) and wet-dog shake responses (Bedard and Pycock, 1977), suggesting that these responses to stimulation of 5-HT_2A_ receptors are good behavioral markers for hallucinations in animals. In normal mice, oral administration of YKS reduced head-twitch behavior induced by treatment with the 5-HT_2A_ receptor agonist 2,5-dimethoxy-4-iodoamphetamine (DOI) (Egashira et al., 2008). In stressed mice, isolation stress for 6 weeks aggravated the hallucination-like behavior, and YKS reversed this aggravation, which was thought to be mediated by additive and/or synergic effects of Bupleurum root, Uncaria hook, Japanese Angelica root, and Glycyrrhiza in YKS (Ueki et al., 2015b). A similar result was seen in rats subjected to ischemia/reperfusion injury (Nogami et al., 2011).

#### Anxiety

Oral YKS ameliorated anxiety-like behavior in normal mice (Kamei et al., 2009; Shoji and Mizoguchi, 2013) and aged rats (Mizoguchi et al., 2010b) in the elevated-plus maze. Similar effects have also been demonstrated in rats subjected to cerebrovascular ischemia in both light/dark box and elevated-plus maze tests (Nogami et al., 2011), and fear-conditioned rats during re-exposure to the context (Yamaguchi et al., 2012). In addition, YKS ameliorated abnormal behavior in APP-Tg mice, as shown by increased number of entry and prolonged time in the open arm of an elevated-plus maze, suggesting that YKS ameliorates disinhibition in APP-Tg mice (Tabuchi et al., 2009).

#### Sleep Disturbance

Yokukansan has also been shown to reverse the shortening of sleep time induced by pentobarbital injection in socially isolated mice (Egashira et al., 2011). Recently, using electroencephalography and electromyography, Nagao et al. (2014) investigated the effect of oral administration of YKS on sleep disturbance in a rat model of cerebrovascular dementia. Rats with cerebral ischemia had a higher total wakefulness time and lower total non-rapid eye movement sleep, so it was concluded that sleep disturbances were ameliorated by YKS. More recently, Ogawa et al. (2016) examined the sleep-promoting effect of YKS by thermography, which evaluates the decrease in skin temperature of mice during sleep. The authors showed that YKS effectively decreased skin temperature. This result suggests sleep-promoting effect of YKS. They suggested direct effects of Uncaria hook, Bupleurum root, Cnidium rhizome, and Japanese Angelica root, together with indirect effects of Poria sclerotium, Atractylodes Lancea rhizome, and Glycyrrhiza, for the sleep-promoting effect of YKS.

#### Hyperlocomotion

Yokukansan has no effect on motor activity in general (Ikarashi et al., 2009; Kanno et al., 2009; Tabuchi et al., 2009; Mizoguchi et al., 2010b, 2011; Fujiwara et al., 2011; Nishi et al., 2012). However, it has been reported to mitigate hyperlocomotor activity in mice treated with methamphetamine (Makinodan et al., 2009; Uchida et al., 2009) and in gerbils subjected to ischemia/reperfusion injury (Liu et al., 2014).

#### Maladaptation

The effect of YKS on the adaptive response was examined by using an automatic hole-board test in chronically stressed mice (Tsuji et al., 2014). Thus, a restraint stress for 60 min decreased the number of head-dipping behavior, which means increased emotional response. However, this was not observed in mice exposed to repeated stress for 14 days, suggesting the development of adaptation by repeating stress for 60 min. However, mice exposed to 240 min-stress for 14 days did not show adaptation to the stress. This maladaptation was ameliorated by YKS or 5-HT_1A_ receptor agonist flesinoxan. These findings suggest that YKS may facilitate stress adaptation via alleviation of emotional abnormality under conditions of excessive stress.

#### Prepulse Inhibition

A weaker prepulse is known to inhibit a subsequent stronger stimulus pulse. This phenomenon is generally referred to prepulse inhibition (PPI), which is the temporarily adaptive response to a stronger startle-inducing sensory stimulus when preceded by a weaker “warning” signal (Makinodan et al., 2009). In the pathophysiology, reduced PPI (a startle response similar to that observed without a prepulse) is a behavioral end-phenotype of schizophrenia in humans and in animal models. In the latter, reduced PPI is also observed in pups of dams injected intraperitoneally with polyinosinic:polycytidylic acid (poly I:C), a synthetic analog of double-stranded RNA that mimics the *in utero* response to viral infection, and which indicates a possible etiology of schizophrenia. Oral administration of YKS has been reported to reverse this reduced PPI (Makinodan et al., 2009).

### Effects of YKS on Non-BPSD Symptoms

#### Tardive Dyskinesia

Repeated injection of haloperidol into the thigh induces vacuous chewing movement in rats, which is known as an index of tardive dyskinesia. YKS alleviated haloperidol-induced vacuous chewing movement (Sekiguchi et al., 2012).

#### Neuropathic Pain

Oral YKS improved filament-induced mechanical allodynia and acetone-induced cold allodynia in rats with chronic constriction injury (Suzuki et al., 2012). YKS also improved mechanical allodynia through the regulation of interleukin 6 (IL-6) expression in the spinal cord of mice with neuropathic pain (Ebisawa et al., 2015).

#### Morphine Tolerance and Physical Dependency

An opioid analgesic morphine is often used to treat several types of pain that is related to operation and cancer. It is well-known that the prolonged use can induce analgesic tolerance, physical dependence, and addiction. Nakagawa et al. (2012) demonstrated that YKS alleviated tolerance and physical dependence induced by repeated administration of morphine, without affecting the analgesic effect in mice.

#### Allergy/Atopic Dermatitis

Yokukansan inhibited development of atopic dermatitis-like skin lesions in socially isolated NC/Nga mice by suppression of scratching and grooming behaviors, inhibition of mast cell and eosinophil infiltration, and enhancement of skin moisture retention (Jiang et al., 2009; Funakushi et al., 2011). Yamamura et al. (2014) also reported that YKS inhibited mast cell degranulation, TNFα release from mast cell-like RBL-2H3 basophil leukemia cells, and TNFα-induced intercellular adhesion molecule-1 expression in human endothelial cells. These ameliorative effects were not the actions on central nervous system, but were thought to be indirectly mediated by the psychopharmacological effects of YKS such as the anxiolytic effect (Yamamura et al., 2014).

### Effects of YKS on Cognitive Dysfunction

Yokukansan prevented cognitive disturbances in APP-Tg mice (Tabuchi et al., 2009; Fujiwara et al., 2011), mice with intracerebroventricular Aβ-injection (Sekiguchi et al., 2011), rats subjected to cerebrovascular ischemia (Nogami et al., 2013) or intracerebroventricular Aβ injection with ischemia/reperfusion injury (Uchida et al., 2013), gerbils with cerebral ischemia (Liu et al., 2014) and rats with thiamine-deficiency (Ikarashi et al., 2009). YKS was also shown to ameliorate cognitive deficits in animal models of schizophrenia, such as poly I:C-injected mice (Makinodan et al., 2009), Gunn rats (Furuya et al., 2013), and aged rats (Mizoguchi et al., 2011).

## Brain Regions Responsible for the Psychopharmacological Effects of YKS

### Cerebral Cortex

Areas not in sensory or motor areas of the cerebral cortex are termed “association areas,” and are considered to integrate higher cerebral functions such as cognition, judgment, memory, language, and dense exercise. There are known parietal and temporal association areas, and a frontal association area, the latter of which includes the prefrontal cortex (PFC). The PFC of rodents consists of several areas, including the prelimbic and infralimbic cortexes, which are thought to be involved in cognitive performance and emotional responses (Shoji and Mizoguchi, 2013).

Orally administered YKS has been suggested to affect the cerebral cortex. For example, histopathological examination showed that YKS inhibited the concomitant degeneration of neuronal and astroglial cells in the cerebral cortexes of thiamine-deficient rats, as produced by a thiamine-deficient diet for 37 days; in that study, YKS ameliorated BPSD-like symptoms such as aggressiveness and anxiety (Ikarashi et al., 2009). *In vitro* primary culture studies using rat cortical astrocytes suggest that YKS ameliorates the thiamine-deficient-induced dysfunction of glutamate transport into astrocytes via increased expression of glutamate transporters (Kawakami et al., 2009, 2010). In primary cultured cortical neurons, YKS also inhibited glutamate-induced neuronal death (Kawakami et al., 2011).

Several reports have demonstrated that the efficacy of YKS is associated with its effects in the PFC. In socially isolated mice, dietary supplementation with YKS increased the 5-HT_1A_ receptor density in the PFC, which was thought to mediate augmentation of behavioral response (i.e., decreased rearing behavior) to the 5-HT_1A_ receptor agonist (±)-8-hydroxy-2-(dipropylamino)tetralin hydrobromide (8-OH-DPAT) (Ueki et al., 2015a). YKS has a partial agonistic action for 5-HT_1A_ receptors (Terawaki et al., 2010), and geissoschizine methyl ether (GM), an indole alkaloid derived from Uncaria hook, has been identified as the active ingredient responsible for the partial agonism of 5-HT_1A_ receptors by YKS (Nishi et al., 2012). Although speculative, the up-regulatory effect of YKS on prefrontal 5-HT_1A_ receptors leads us to hypothesize that YKS has the actions to increase the target molecule(s) and stimulate it for the effective amelioration of psychological symptoms such as aggressiveness and anxiety.

The relationship between YKS and PFC has also been demonstrated in 5-HT_2A_ receptors. YKS has been shown to downregulate 5-HT_2A_ receptor expression in the PFCs and reduce the DOI-induced hallucination-like behavior (head-twitching) in normal mice (Egashira et al., 2008) and stressed mice (Ueki et al., 2015b). It has been suggested that there is a synergistic effect of Bupleurum root, Uncaria hook, Japanese Angelica root, and Glycyrrhiza among seven constituent components of YKS is suggested to be concerned in this downregulation of 5-HT_2A_ receptors (Ueki et al., 2015b). These results indicate that the reduction of DOI-induced hallucination-like behavior by YKS might involve down-regulation of 5-HT_2A_ receptors in the PFC. Taken together, the improving effects of YKS on aggressive, hallucination-like, and anxiety-like behaviors might be mediated by acting together with the effects on 5-HT_1A_ and 5-HT_2A_ receptors in the PFC region.

In the PFC of aged rats, a decrease in the proliferation and migration of neuronal stem/progenitor cells identified by incorporation of bromodeoxyuridine was observed (Tanaka and Mizoguchi, 2009). In addition, this decrease might be caused by changes in a microenvironment formed by extracellular matrix, because increased expression of aggrecan, a major molecule of chondroitin sulfate proteoglycans, which are thought to inhibit axonal growth, migration, and synaptic plasticity of neurons (Perris and Johansson, 1990; Bradbury et al., 2002; Schwartz and Domowicz, 2004), was also observed in the aged PFC. Both the decrease in bromodeoxyuridine-labeled cells and the increase in aggrecan expression in the PFC of aged rats were improved by YKS treatment for 3 months, suggesting that activation of neuroplasticity, including proliferation and migration, is facilitated by the action of YKS in the PFC region.

In addition, the age-related working memory deficit is suggested to be caused by reduced extracellular dopamine levels in the prelimbic region of the PFC (Mizoguchi et al., 2009). A similar causal relation between reversal learning deficit and orbitofrontal regions of the PFC is suggested (Mizoguchi et al., 2010a). YKS improved not only these working memory and reversal learning deficits but also the decrease in dopamine neurotransmission in the PFC structure (Mizoguchi et al., 2010b, 2011), and the improvement of the deficits was counteracted by infusion of a dopamine D_1_ receptor antagonist SCH23390 into these prefrontal cortical regions (Mizoguchi et al., 2011). YKS may interact in the PFC to improve age-related learning and memory deficits. Thus, YKS is thought to be effective for cognitive deficits and obstinacy in aged subjects.

Aged rats also showed anxiety-like behaviors and decreased 5-HT release in the prelimbic region of the PFC, and YKS improved these age-related changes (Mizoguchi et al., 2010a). In other research, Shoji and Mizoguchi (2013) have shown the relationships between the anxiolytic effects of YKS and the PFC. Thus, YKS ameliorated restraint stress-induced anxiety and increased c-Fos expression, a maker of neuronal activation, in the prelimbic cortex of the PFC in young rats. This result supports that the anxiolytic effect of YKS is related to its effects in the prelimbic cortical region of the PFC.

**FIGURE 1:**
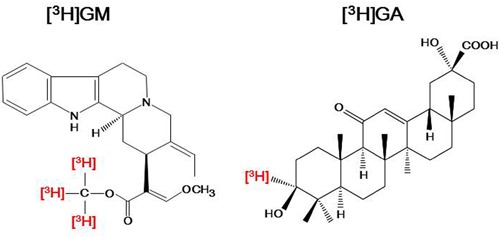
**Chemical structures of tritium-labeled geissoschizine methyl ether ([^3^H]GM) and 18β-glycyrrhetinic acid ([^3^H]GA).** For [^3^H]GM, three tritium atoms were introduced into the methyl group of the methyl ester in its structure; For [^3^H] GA, one tritium atom was introduced at the third position in its structure.

**FIGURE 2:**
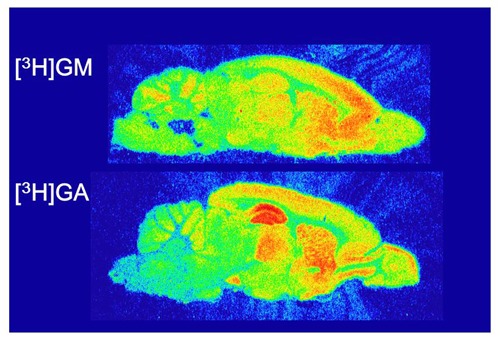
**Autoradiograms to determine specific bindings of tritium-labeled geissoschizine methyl ether ([^3^H]GM) and 18β-glycyrrhetinic acid ([^3^H]GA) in rat brain slices.** Freshly frozen sections of rat brain were reacted with [^3^H]GM or [^3^H]GA and exposed to a tritium-sensitive imaging plate. After exposure, the plates were analyzed in an imaging analyzer to generate autoradiograms. Specific binding sites of [^3^H]GM were densely detected in the frontal cortex and those of [^3^H]GA were in the hippocampus. These findings are cited from the following articles: Mizoguchi et al. (2014a) for [^3^H]GM; Mizoguchi et al. (2014b) for [^3^H]GA.

Studies have indicated that GM reaches the brains of rats given YKS orally by crossing the blood–brain barrier (BBB) (Imamura et al., 2011; Kushida et al., 2013). In the autoradiography analysis using tritium-labeled GM ([^3^H]GM) (**Figure 1**), we identified that the frontal cortex, including the PFC, had the highest density of specific binding for [^3^H]GM (**Figure 2**) (Mizoguchi et al., 2014b). Candidate molecules of the binding sites were identified by competition-binding assay, and included 5-HT_1A_, 5-HT_2A_, 5-HT_2B_, 5-HT_2C_, 5-HT_7_, D_2_, adrenergic α_2A_, and μ-opioid receptors, and L-type Ca^2+^ channels. A similar result has been obtained in a single-cell-based calcium imaging assay (Ueda et al., 2011). Moreover, the [^3^H]GM signals were accumulated in frontal cortical region in the microautoradiography analysis, including neuron-like large cells. Taken together, these findings suggest that GM specifically binds to the frontal cortex of rats, and that GM expresses its several pharmacological actions through 5-HT receptors in the frontal cortex.

### Limbic System

The limbic system is a generic term for a region that includes the paleocortex, archipallium, mesocortex, and subcortical nuclei. Here we include the amygdala, hippocampus, and corpus callosum, which are related to emotion (delight, anger, sorrow and pleasure) and short- and long-term memory.

#### Amygdala

Shoji and Mizoguchi (2013) showed that restraint stress induces anxiety state and the increase in c-Fos expression in the basolateral and medial amygdaloid nuclei in rats. YKS suppressed these stress-induced phenotypes, suggesting that these areas are related to the anxiolytic effect of YKS.

#### Hippocampus

Neuronal and astroglial degenerations, as described in Section “Effects of YKS on BPSD-Like Symptoms,” were also observed in the hippocampus of thiamine-deficient rats, and these degenerations were ameliorated by YKS (Iizuka et al., 2010).

YKS administration for 14 days improved spatial memory impairment induced by combination of intracerebroventricular Aβ injection and ischemia/reperfusion injury in rats (Uchida et al., 2013). This effect may be mediated by increasing acetylcholine release via the regulation of dynamin 1 in the hippocampus. The same regimen of YKS also improved spatial memory disturbance, reduced acetylcholine release, and neuronal apoptosis in the hippocampal CA1 subfield of rats subjected to ischemia/reperfusion injury (Nogami et al., 2013). Oral administration of YKS for 30 days also ameliorated not only locomotor hyperactivity and memory impairment but also hippocampal CA1 neuronal death, inflammatory response, and oxidative DNA damage induced by ischemia/reperfusion injury in gerbils (Liu et al., 2014), suggesting that YKS inhibits the inflammatory response, oxidative stress, and subsequent neuronal death in the hippocampus induced by transient ischemia. YKS has also been suggested to prevent cognitive deficits through suppression of microglia activation and promotion of neurogenesis in the hippocampal dentate gyrus of Gun rats, an established animal model of schizophrenia (Furuya et al., 2013).

In zinc-deficient rats, a model of neurological disease, YKS attenuated the increased extracellular glutamate concentration in the hippocampus (Takeda et al., 2008). As the underlying mechanisms, glutamate release from the presynaptic terminal in the hippocampal CA3 subfield was increased by tetanic stimuli at the dentate granule cell layer in the hippocampal slice prepared from zinc-deficient rat, compared with controls. This increase was attenuated in the preparations from rats orally administered YKS (Takeda et al., 2008).

In other research, pretreatment with oral YKS 1 h before stress exposure was demonstrated to normalize the stress-induced elevation of plasma corticosterone, a stress hormone that is cytotoxic toward neurons (Shimizu et al., 2015a,b). Corticosterone-induced cytotoxicity of mouse embryonic hippocampal neurons is also reported to be protected by treatment with YKS (Nakatani et al., 2014).

Decreased proliferation and migration of neuronal stem cells and increased expression of aggrecan were also observed in the hippocampus of aged rats, and YKS was shown to improve these changes (Tanaka and Mizoguchi, 2009). The promoting effect of YKS was also demonstrated in oligodendrocytes, where YKS promoted the proliferation and differentiation of oligodendrocytes in an experiment that used purified mouse oligodendrocyte precursor cells. GM was identified as the active ingredient responsible for this effect (Ueki et al., 2014). Elsewhere, this *in vitro* effect has been supported in *in vivo* research by Morita et al. (2014), who examined the effect of GM on demyelination in cuprizone-treated mice. They demonstrated that GM significantly increased bromodeoxyuridine-labeled GSTpi^+^ mature oligodendrocytes and reversed the decrease in myelin basic protein immunoreactivity. These results indicate that GM is likely to promote the proliferation of oligodendrocytes and their differentiation into mature oligodendrocytes.

It is known that Glycyrrhiza-derived glycyrrhizin is metabolized to 18β-glycyrrhetinic acid (GA) by β-glucuronidase activity in the enteric bacteria. This metabolite is absorbed into the systemic circulation, then distributes to the brain (Tabuchi et al., 2012). Given that several *in vitro* studies suggest that GA is a potent active ingredient, we examined whether GA specifically binds to the brain tissue using [^3^H]GA (**Figure 1**). Autoradiography found that the hippocampus (including the dentate gyrus) is the highest density of specific binding for [^3^H]GA (**Figure 2**) (Mizoguchi et al., 2014a). The candidate molecules of the binding sites were not steroid receptors, gap junctions, glutamate transporters, and glutamate receptors. Moreover, in the microautoradiography analysis, [^3^H]GA distributed in hippocampal astrocyte-like cells containing 11β-hydroxysteroid dehydrogenase type-1 (11β-HSD1, Mizoguchi et al., 2014a). These results suggest that GA specifically binds to the hippocampus of rats, and that GA expresses its several pharmacological actions through 11β-HSD1 in astrocytes.

#### Corpus Callosum

The corpus callosum connects the right and left cerebral hemisphere. The neural fibers contained in it facilitate interhemispheric cerebral communication. In adult mice, acute stress increased corticosterone in the plasma and decreased glucocorticoid receptor (GR) protein, but did not change its mRNA expression in oligodendrocytes of the corpus callosum (Shimizu et al., 2015b). MicroRNAs (miRs) are non-coding RNAs that inhibit the translation and/or decrease the stabilities of their target mRNAs, ultimately decreasing target protein expression (Carthew, 2006; Wang et al., 2007). MiRs also regulate development and differentiation of neurons (Stefani and Slack, 2008; Chiu et al., 2014). MiR-124a is a candidate negative regulator of GR expression in the brain (Vreugdenhil et al., 2009), and Shimizu et al. (2015b) showed that acute stress increased its expression in oligodendrocytes of the corpus callosum. In a drug treatment study, YKS normalized the stress-induced changes in plasma corticosterone, GR protein, and miR-124a expression, although GR mRNA level was not significantly changed. Thus, YKS may alleviate the stress-induced decrease in GR protein by downregulating miR-124a expression.

### Basal Ganglia

Generally, the term basal ganglia is applied to the caudate nucleus, putamen, and globus pallidus, and functionally related to the substantia nigra and subthalamic nucleus. The caudate nucleus and the putamen are called the striatum (Ikarashi and Maruyama, 1999).

#### Striatum

The vacuous chewing movement in rats induced by haloperidol can be ameliorated by oral administration of YKS (Sekiguchi et al., 2012). Real-time PCR and microdialysis analyses showed that YKS increased mRNA expression of an astroglial glutamate transporter, glutamate transporter 1 in the striatum, and decreased haloperidol-evoked elevation of striatal glutamate release. This result suggests that the amelioration by YKS against vacuous chewing involved the inhibition of excessive extracellular glutamate through the facilitation of striatal glutamate transporter expression.

#### Nigrostriatum

Unilateral nigrostriatal lesions by microinjecting 6-hydroxydopamine (6-OHDA) into the rat medial forebrain bundle decreased tyrosine hydroxylase immunoreactivity (i.e., dopamine loss) in the striatum, which has been established as an animal model of Parkinson’s disease (Ishida et al., 2016). Repeated intraperitoneal injection of L-3,4-dihydroxyphenylalanine (L-DOPA) to the 6-OHDA-lesioned rats induced abnormal rotational and axial movements. YKS enhanced these abnormal movements. This might be due to the augmentation of dopamine supplementation via inhibition of catechol-*O*-methyltransferase in the striatum. GM and corynoxeine, which are alkaloids in Uncaria hook, are suggested to contribute to the inhibitory effects of YKS (Ishida et al., 2016).

Doo et al. (2010) investigated the neuroprotective effects of Korean Yi-Gan San (YGS), which is composed of nine medical herbs (the seven medical herbs used in YKS in Japanese Kampo, plus Ponciri fructus and Magnoliae cortex). They used a model of *in vitro* and *in vivo* 1-methyl-4-phenylpyridine (MPP^+^)/1-methyl-4-phenyl-1,2,3,6-tetrahydropyridene (MPTP)-induced cytotoxicity, and showed that pretreatment of SH-SY5Y human neuroblastoma cells with Korean YGS protected against MPP^+^-induced cell degeneration and decreased caspase-3 activity concomitant with increased phosphorylated Akt expression. LY294002, the inhibitor of phosphatidyl-inositol 3-kinase (PI3K)/Akt, reduced the effectiveness of Korean YGS. In MPTP-treated mice, Korean YGS treatment regained abnormal movement, and also inhibited nigrostriatal dopaminergic neuronal loss. From these results, it was concluded that Korean YGS can rescue dopaminergic degeneration from MPP^+^/MPTP cytotoxicity through PI3K/Akt signaling activation. A similar effect of YKS on the PI3K/Akt pathway has been demonstrated (Kubota et al., 2013). Thus, YKS and Korean YGS might each prevent dopaminergic neuronal loss in the nigrostriatal region.

### Diencephalon

The diencephalon including the thalamus and hypothalamus plays a role related to various vital functions. In thiamine-deficient rats used to assess the effects of YKS on BPSD-like behaviors, the extracellular glutamate concentration was increased in vulnerable brain regions, including the thalamus (Langlais and Zhang, 1993). In the ventral posteromedial thalamus, this elevation was normalized by oral administration of YKS (Ikarashi et al., 2009), indicating that YKS might affect the thalamus. Furthermore, GR signaling has been known to regulate the hypothalamic–pituitary–adrenal axis activity. The axis activation also affects GR expression. YKS normalized a stress-induced decrease in GR protein in the paraventricular nucleus (Shimizu et al., 2015a,b). YKS also decreased miR-18, another negative regulator for GR expression in the paraventricular nucleus, although GR mRNA levels were not significantly changed (Shimizu et al., 2015a,b). These results suggest that YKS normalizes the stress-induced decrease in GR protein in the paraventricular nucleus by downregulating miR-18 levels and thereby disinhibiting mRNA translation.

### Brainstem

The brainstem includes the medulla oblongata, pons, and midbrain, and research has examined the effects of YKS on morphological damage in the medulla-pons of thiamine-deficient rats. Sponge-like degeneration was detected in this area by light microscopy (Ikarashi et al., 2009), and the neuronal and astroglial degenerations were detected in the vestibular nucleus by electron-microscopy (Iizuka et al., 2010). Both changes were inhibited by treating YKS.

Nakagawa et al. (2012) have reported that repeated morphine treatment causes α2A-adrenoceptor reduction in the medulla-pons of mice, and this was prevented by Glycyrrhiza and Uncaria hook, as well as YKS. The α2A-adrenoceptor antagonist yohimbine showed similar effects to YKS. Moreover, YKS, Glycyrrhiza, and Uncaria hook had antagonistic effects on the α2A-adrenoceptors. These results suggested that Glycyrrhiza and Uncaria hook in YKS inhibit tolerance and physical dependence induced by repeated administration of morphine in mice (described above) through their ability to block α2A-adrenoceptors, thereby preventing decreased membrane expression of the receptors in the medulla-pons. Glycyrrhiza-derived GA and Uncaria hook-derived GM are thought to be the active ingredients responsible for the efficacy of YKS.

### Spinal Cord

Single administration of YKS has been reported to inhibit allodynia in rats subjected to chronic constriction injury (Suzuki et al., 2012). *In vivo* microdialysis showed that YKS inhibited brush- or acetone-induced increases in glutamate concentrations in the cerebrospinal fluid, and the antiallodynic actions of YKS were counteracted by intrathecal injection of the glutamate transporter inhibitor, suggesting that the antiallodynic actions are associated with attenuation of glutamate neurotransmission via glutamate transporter activation in the spinal cord. We demonstrated that GA was responsible for the ameliorating effect of YKS on glutamate transport dysfunction via glutamate transporter activation in astrocytes (Kawakami et al., 2009, 2010), and also that GA was detected in the cerebrospinal fluid of rats administered YKS orally (Tabuchi et al., 2012). Thus, the analgesic effect of YKS may be from inhibition of pain transmission to the upper central nervous system through the suppression of excess glutamate release from primary afferent fibers via glutamate transporter activation in astrocytes of the spinal cord.

In partial sciatic-nerve ligated mice, Ebisawa et al. (2015) showed that YKS relieved mechanical allodynia and inhibited IL-6 mRNA expression (a mediator of pain) in the spinal cords. These results indicated that the anti-nociceptive action of YKS might be mediated by suppressed IL-6 mRNA expression in spinal astrocytes and microglias. They suggest that Atractylodes lancea rhizome may be the active component.

## Conclusion

**FIGURE 3:**
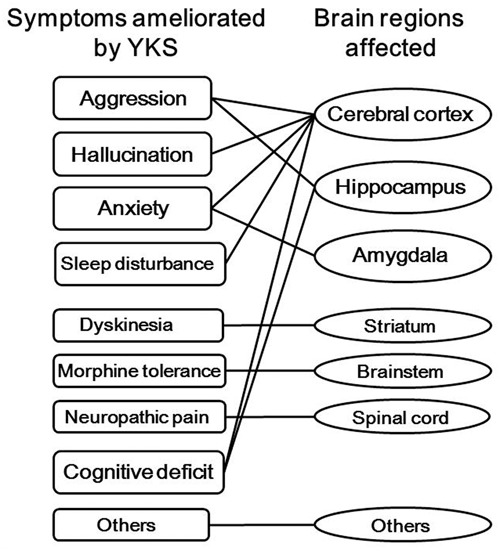
**Multiple psychopharmacological effects of yokukansan (YKS) and the brain regions affected.** YKS consists of seven medicinal herbs, so has ameliorative effects on aggression, hallucination, anxiety, and so on, which are related to the specific brain regions. Other effects include amelioration of hyperlocomotion, maladaptation, and reduced prepulse inhibition. Other brain regions include the corpus callosum, thalamus, and hypothalamus.

Dementia is a most problematic age-related neurodegenerative disorder of modern society, with a prevalence exceeding 4.62 million people in Japan. Dementia is often complicated by BPSD, and many clinical trials have shown the usefulness of YKS for the treatment of related aggressiveness, agitation, and hallucination, without producing severe side effects. However, several adverse drug reactions have been reported; they occurred in 136 cases (4.3%) in total among 3,156 patients treated with YKS (Hisada et al., 2014). In addition, pseudohyperaldosteronism should be paid attention because YKS contains Glycyrrhiza. In parallel with clinical trials, basic research can now explain several of the neuropsychopharmacological effects of YKS. In the present review, we described the multiple psychopharmacological effects of YKS, including the ameliorative effects on aggressiveness, hallucination, anxiety, sleep disturbance, hyperlocomotion, maladaptation, reduced PPI, tardive dyskinesia, neuropathic pain, morphine dependence, allergy/atopic dermatitis, and cognitive dysfunction in experimental animals. Following this, we attempted to identify evidence for the relationship between the psychopharmacological effects and the brain regions that are related to the effects, which showed that YKS affected the cerebral cortex, hippocampus, striatum, thalamus, hypothalamus, corpus callosum, and spinal cord, which are all involved in behavioral, psychiatric, cognitive, and perceptional dysfunctions (**Figure 3**). Given that YKS is composed of seven medicinal herbs that contain a multitude of ingredients, these findings suggest that its psychopharmacological effects might be attributable to multiple rather than individual brain regions. Among the active ingredients of YKS, GM and GA seem to predominate (**Figures 1**–**3**). GM, which comes from Uncaria hook, is suggested to act predominantly in the frontal cortical regions of the cerebral cortex, where it behaves as a partial agonist of 5-HT_1A_ receptors, and thereby mediates the anti-aggressive and anxiolytic effects of YKS. GA, which is the major metabolite of glycyrrhizin in Glycyrrhiza, is suggested to act predominantly in the hippocampus, where it might enhance glutamate uptake into astrocytes via activation of glutamate transporters.

Brain function is regulated or controlled by a complex neural network that involves several neurotransmitter pathways, with glutamate, 5-HT, noradrenaline, dopamine, and GABA in the particular brain regions, all mediating neuronal signals (Cooper et al., 1991; Ikarashi and Maruyama, 1999). Recently, we reviewed the basic research into YKS, including its multiple neuropharmacological mechanisms related to glutamatergic, serotonergic, cholinergic, dopaminergic, adrenergic, and GABAergic neurotransmission; the ingredients active on those neurotransmitter systems; their pharmacokinetics; and the possible mechanisms of action of YKS (Ikarashi and Mizoguchi, 2016). As described in the present review, various brain regions would no doubt be affected by oral administration of YKS. Although it remains unknown whether the psychopharmacological effects of YKS reflect active brain areas directly or indirectly, our two reviews into YKS should help facilitate a more holistic understanding of the mechanisms underlying its clinical efficacy. To obtain more detail about the beneficial effects of YKS, future research aims to identify the active ingredients and study their additive and/or synergistic effects in the neural network. Currently, it appears that at least GM and GA act in the cerebral cortex and hippocampus, respectively, where they contribute to the observed beneficial effects of YKS (Mizoguchi et al., 2014a,b). In conclusion, this review provides fundamental information about the multiple psychopharmacological effects of YKS and their related brain regions, which should help inform clinical practice and guide future research.

## Author Contributions

KM and YI cooperatively corrected the findings, and prepared the manuscript.

## Conflict of Interest Statement

The authors are employees of Tsumura & Co. The authors declare that, except for income received from the employer, no financial support or compensation has been received from any individual or corporate entity and no conflict of interest exists.
